# O-1602 Promotes Hepatic Steatosis through GPR55 and PI3 Kinase/Akt/SREBP-1c Signaling in Mice

**DOI:** 10.3390/ijms22063091

**Published:** 2021-03-17

**Authors:** Saeromi Kang, Ae-Yeon Lee, So-Young Park, Kwang-Hyeon Liu, Dong-Soon Im

**Affiliations:** 1 College of Pharmacy, Pusan National University, Busan 46241, Korea; saeromi85@gmail.com (S.K.); dodo3239@naver.com (A.-Y.L.); 2 BK21 FOUR KNU Community-Based Intelligent Novel Drug Discovery Education Unit, College of Pharmacy and Research Institute of Pharmaceutical Sciences, Kyungpook National University, Daegu 41566, Korea; soyoung561@hanmail.net (S.-Y.P.); dstlkh@gmail.com (K.-H.L.); 3 Department of Biomedical and Pharmaceutical Sciences, College of Pharmacy, Graduate School, Kyung Hee University, Seoul 02447, Korea

**Keywords:** lysophosphatidylinositol, GPR55, hepatocytes, steatosis, non-alcoholic fatty liver disease

## Abstract

Non-alcoholic fatty liver disease is recognized as the leading cause of chronic liver disease. Overnutrition and obesity are associated with hepatic steatosis. G protein-coupled receptor 55 (GPR55) has not been extensively studied in hepatic steatosis, although its endogenous ligands have been implicated in liver disease progression. Therefore, the functions of GPR55 were investigated in Hep3B human hepatoma cells and mice fed high-fat diets. O-1602, the most potent agonist of GPR55, induced lipid accumulation in hepatocytes, which was reversed by treatment with CID16020046, an antagonist of GPR55. O-1602 also induced intracellular calcium rise in Hep3B cells in a GPR55-independent manner. O-1602-induced lipid accumulation was dependent on the PI3 kinase/Akt/SREBP-1c signaling cascade. Furthermore, we found increased levels of lysophosphatidylinositol species of 16:0, 18:0, 18:1, 18:2, 20:1, and 20:2 in the livers of mice fed a high-fat diet for 4 weeks. One-week treatment with CID16020046 suppressed high-fat diet-induced lipid accumulation and O-1602-induced increase of serum triglyceride levels in vivo. Therefore, the present data suggest the pro-steatotic function of GPR55 signaling in hepatocytes and provide a potential therapeutic target for non-alcoholic fatty liver disease.

## 1. Introduction

Non-alcoholic fatty liver disease is recognized as the leading cause of chronic liver disease. Overnutrition and obesity are associated with non-alcoholic hepatic steatosis, a condition characterized by extensive lipid accumulation in hepatocytes [1]. Lysolipids have been implicated in human obesity and liver disease progression. For example, postprandial lysophospholipid was shown to suppress hepatic fatty acid oxidation in group 1B phospholipase A2-deficient mice [2]. In addition, loss of the membrane bound O-acyltransferase domain-containing 7 gene was associated with the accumulation of its substrate lysophosphatidylinositols, and direct administration of lysophosphatidylinositols promoted hepatic inflammatory and fibrotic transcriptional changes [3]. Levels of lysophosphatidylinositol species such as 16:0, 18:0, and 20:4 have been found to increase in obesity and correlate with weight and fat percentage [4]. G protein-coupled receptor 55 (GPR55) is now recognized as a receptor for lysophosphatidylinositols [5,6] and its expression has been described in the liver of humans and mice [4,7]. However, the role of GPR55 in liver diseases such as hepatic steatosis has not been much studied. Therefore, the functions of GPR55 were investigated in human Hep3B hepatoma cells and in mice fed a high-fat diet using O-1602, the most potent agonist of GPR55, and CID16020046, an antagonist of GPR55 [6,8]. We also measured levels of lysophosphatidylinositol species in the serum and liver from mice fed a high-fat diet for 4 weeks. We found that GPR55 signaling in hepatocytes may promote lipid accumulation in the liver.

## 2. Results 

### 2.1. O-1602 Induces Lipid Accumulation through GPR55 in Hep3B Cells

**Figure 1:**
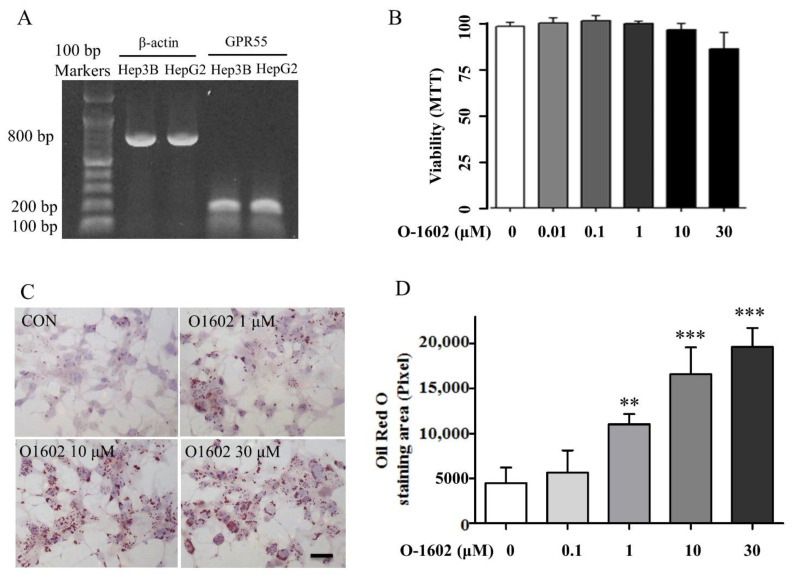
Expression of GPR55 and O-1602-induced lipid accumulation in Hep3B cells. (**A**) GPR55 RT-PCR results in Hep3B and HepG2 cells. (**B**) Viability test of O-1602 in Hep3B cells. (**C**) Oil red O staining. Hep3B cells were treated with different concentrations of O-1602 for 48 h. The red staining shows lipid droplets. The scale bar means 20 μm. (**D**) Histogram showing lipid accumulation. Oil red O staining was analyzed using ImageJ software (NIH, Bethesda, MD, USA). Data are from three individual experiments and expressed as mean ± SD. ** *p* < 0.01, *** *p* < 0.001, compared with the non-treated group.

First, we assessed the expression of GPR55 in human hepatoma cell lines Hep3B and HepG2. GPR55 was detected in both Hep3B and HepG2 cells (Figure 1A). Thus, O-1602, the most potent synthetic GPR55 agonist, was used to test the role of GPR55 in lipid accumulation [6]. No significant cytotoxicity was observed in Hep3B cells treated with O-1602 up to 30 μM (Figure 1B). O-1602 treatment strongly increased the size and number of lipid droplets in Hep3B cells (Figure 1C). O-1602 treatment induced lipid accumulation in a concentration-dependent manner (Figure 1D).

**Figure 2:**
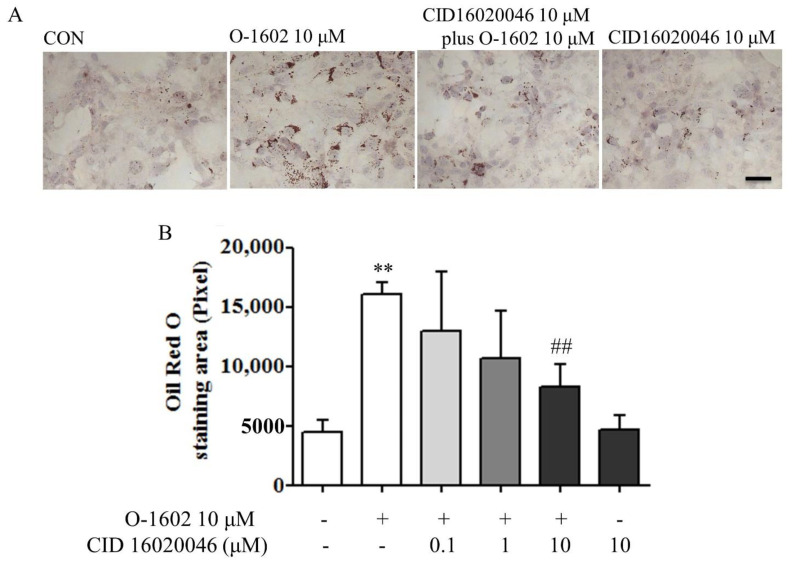
CID16020046 antagonizes O-1602-induced lipid accumulation. (**A**) Oil red O staining. Hep3B cells were pretreated with different concentrations of CID16020046 for 30 min and then treated with 10 μM O-1602 for 48 h. The red staining shows lipid droplets. The scale bar means 20 μm. (**B**) Histogram showing lipid accumulation. Oil red O staining was analyzed using ImageJ software. Data are from three individual experiments and expressed as mean ± SD. ** *p* < 0.01, compared with the non-treated group; ## *p* < 0.01, compared with O-1602-treated group.

CID16020046, a selective antagonist of GPR55, was used to verify whether the pro-lipogenesis effect of O-1602 is mediated by GPR55 [8]. As shown in Figure 2A,B, CID16020046 significantly inhibited O-1602-induced lipid accumulation in a concentration-dependent manner.

### 2.2. O-1602 Induces Intracellular Ca^2+^ Increase in a GPR55-Independent Manner in Hep3B Cells

**Figure 3:**
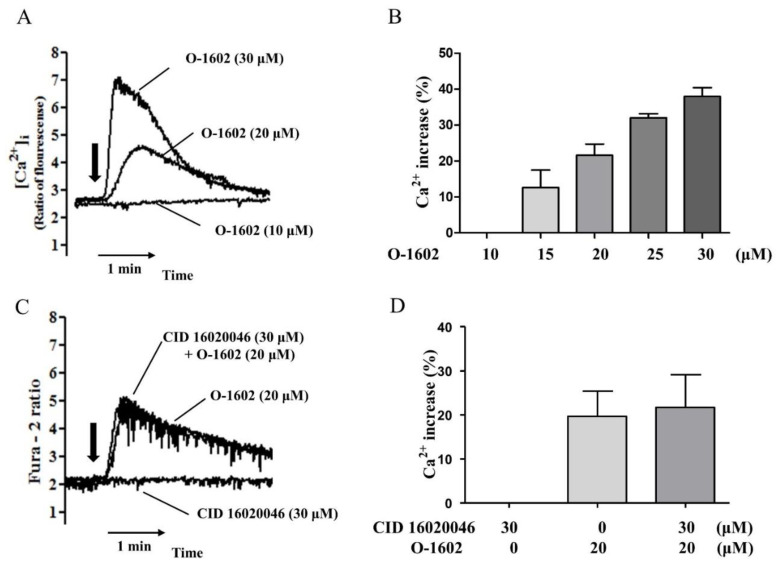
O-1602 induces intracellular Ca^2+^ rise in Hep3B cells. (**A**) Representative Ca^2+^ traces with 10 μM, 20 μM, and 30 μM of O-1602. O-1602 was added at the time point indicated by the arrow. (**B**) Quantitated histogram of O-1602-induced Ca^2+^ increase in Hep3B cells. (**C**) Representative Ca^2+^ traces with 20 μM of O-1602, 30 μM of CID16020046, or both. (**D**) Quantitated histogram of O-1602 and CID16020046-induced Ca^2+^ rise in Hep3B cells. Data are from three individual experiments and expressed as mean ± SD. The maximum Ca^2+^ increase by each treatment was converted to a percentage of the digitonin-mobilized Ca^2+^ increase.

Second, we evaluated the effects of O-1602 on intracellular Ca^2+^ levels in Hep3B (Figure 3A). O-1602 treatment induced an increase in intracellular Ca^2+^ levels in a concentration-dependent manner from 15 μM (Figure 3B). However, CID16020046 pretreatment did not inhibit the O-1602-induced Ca^2+^ increase (Figure 3C,D), suggesting GPR55-independent signaling of O-1602 for the Ca^2+^ response in Hep3B cells.

### 2.3. PI3K/Akt Signaling in the O-1602-Induced Lipid Accumulation in Hep3B Cells

**Figure 4:**
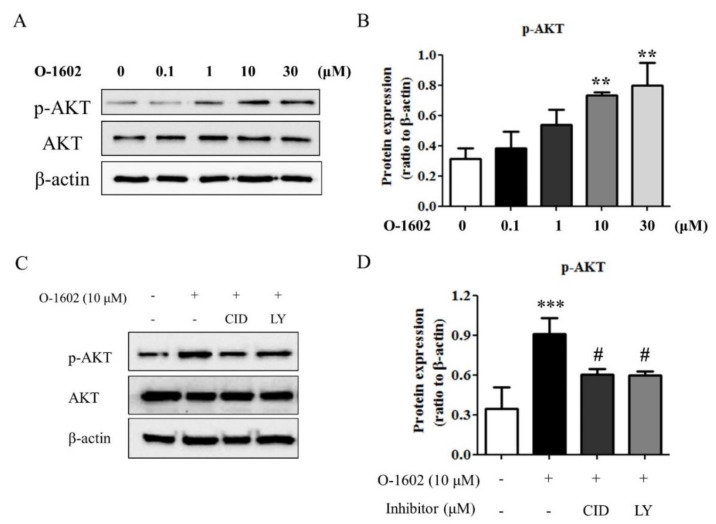
O-1602 induces activation of Akt through GPR55 and PI3K. Hep3B cells were treated with different concentrations of O-1602 for 48 h. Western blotting analysis was performed for Akt and phospho-Akt (S347) in Hep3B cells. (**A**) Representative blots of phospho-Akt, Akt, and β-actin. (**B**) Quantitative western blot analysis of phospho-Akt. Hep3B cells were pretreated with CID16020046 or LY294002 for 30 min and then treated with 10 μM O-1602 for 48 h. (**C**) Representative blots of phospho-Akt, Akt, and β-actin. (**D**) Quantitative western blot analysis of phospho-Akt. Data are from three individual experiments and expressed as mean ± SD. ** *p* < 0.01, *** *p* < 0.001, compared with the non-treated group; # *p* < 0.05, compared with O-1602-treated group.

To investigate the signaling pathway of the O-1602-GPR55 response, phosphorylation of serine 374 of Akt was measured by western blot analysis as an index of Akt activation. As shown in Figure 4A,B, O-1602 induced the phosphorylation of Akt in a concentration-dependent manner. Pretreatment of Hep3B cells with CID16020046 or LY294002, a PI3K specific inhibitor, inhibited the O-1602-inudced phosphorylation of Akt (Figure 4C,D), suggesting the involvement of GPR55 and PI3K in Akt activation by O-1602 treatment.

### 2.4. O-1602 Induces SREBP-1c through GPR55 and PI3K in Hep3B Cells

**Figure 5:**
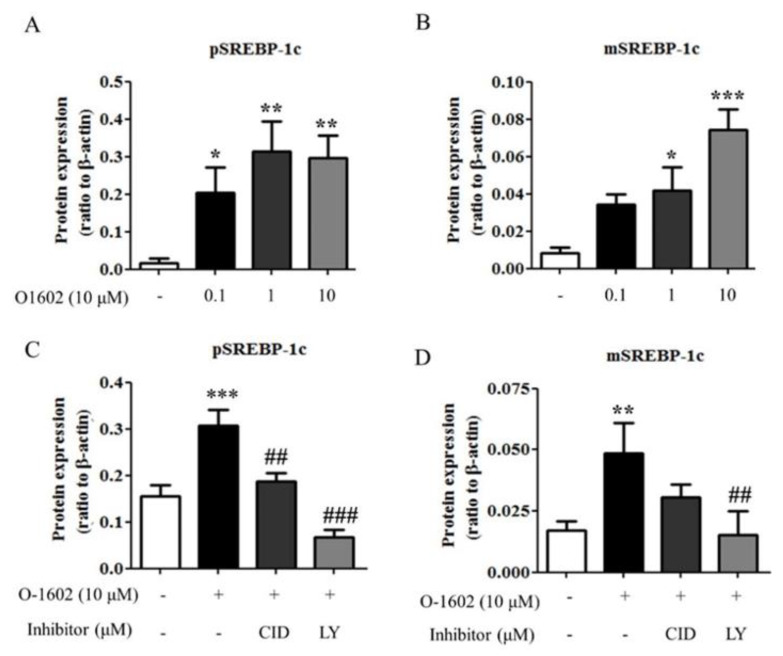
O-1602 induces SREBP-1c protein expression through GPR55 and PI3K. Hep3B cells were treated with different concentrations of O-1602 for 48 h. Western blot analysis was performed for SREBP-1c in Hep3B cells. (**A**,**B**) Quantitative western blot analysis of SREBP-1c preform (**A**) and mature form (**B**). Hep3B cells were pretreated with CID16020046 or LY294002 for 30 min and then treated with 10 μM O-1602 for 48 h. (**C**,**D**) Quantitative western blot analysis of SREBP-1c preform (**C**) and mature form (**D**). Data are from three individual experiments and expressed as mean ± SD. * *p* < 0.05, ** *p* < 0.01, *** *p* < 0.001, compared with the non-treated group; ## *p* < 0.01, ### *p* < 0.001, compared with O-1602-treated group.

SREBP-1c is the main transcription factor for hepatic lipogenic genes in hepatic steatosis [9,10]. Thus, the effects of O-1602 on SREBP-1c expression was evaluated. O-1602 treatment induced the expression of preform and mature form SREBP-1c proteins in a concentration-dependent manner (Figure 5A,B). In addition, the O-1602-mediated induction of SREBP-1c was markedly inhibited by treatment of CID16020046 or LY294002 (Figure 5C,D). Therefore, these data suggest that activation of GPR55 by O-1602 induces the expression of SREBP-1c through PI3K/Akt in hepatocytes, leading to lipid accumulation.

### 2.5. Increase of Lysophosphatidylinosiltol Levels in Livers from High-Fat Diet-Fed Mice In Vivo

**Figure 6:**
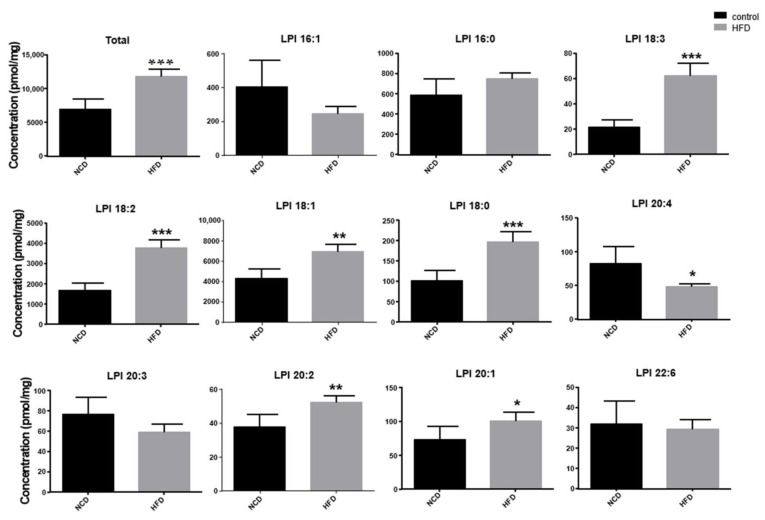
Levels of lysophosphatidylinositols in the livers of mice fed a normal chow diet or high-fat diet. Mice were fed a normal chow diet (NCD) or high-fat diet (HFD) for 4 weeks (*n* = 5). Levels of lysophosphatidylinositols in the liver were measured. Data are expressed as mean ± SD from 5 mice for each group. * *p* < 0.05, ** *p* < 0.01, *** *p* < 0.001, compared with the NCD group.

To determine the in vivo significance of the O-1602-GPR55 response in hepatocytes, we measured the levels of lysophosphatidylinositols in the livers of mice fed a normal chow diet or a high-fat diet for 4 weeks. The levels of lysophosphatidylinositol species of 18:0, 18:1, 18:2, 18:3, 20:1, and 20:2 in the livers of mice fed high-fat diets were significantly higher than those in mice fed normal chow diets (Figure 6, Table 1). Although levels of 20:4 lysophosphatidylinositol were lower in the high-fat diet group, total levels of lysophosphatidylinositols were significantly higher in the high-fat diet group than iin the normal chow diet group (Figure 6). We also measured the levels of lysophosphatidylinositol species in the serum, but there was no significant difference between the two groups (data not shown).

### 2.6. Administration of CID16020046 Reduced High-Fat Diet-Induced Lipid Accumulation in the Liver and O-1602-Induced Increase of Triglycerides Levels in the Serum

**Figure 7:**
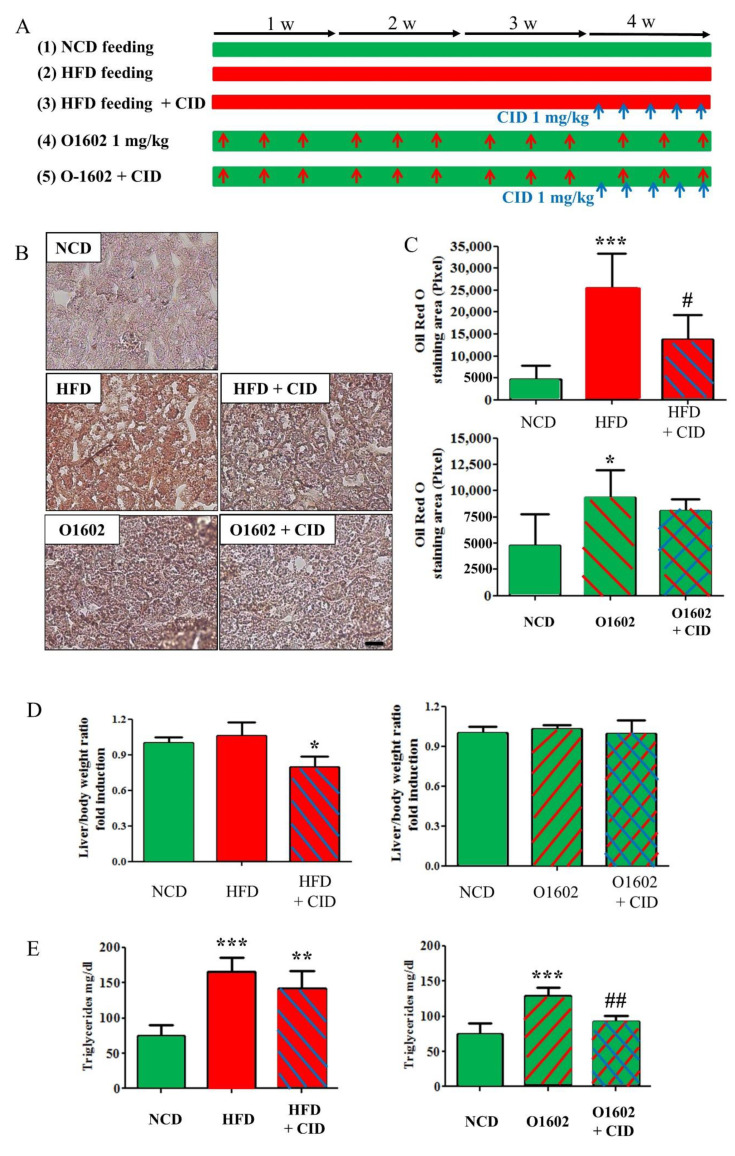
CID16020046 inhibits O-1602-induced triglycerides accumulation in the liver. (**A**) Experimental protocol. HFD was fed to C57BL/6 mice for 4 weeks and O-1602 was administrated by i.p. injection three times per week for 4 weeks. CID16020046 was administrated by i.p. injection five times per week for the last week of 4-week treatment of HFD or O-1602. (**B**) Oil red O staining of liver sections. (**C**) Histograms of oil red O staining. The scale bar means 20 μm. (**D**) Liver/body weight ratio. (**E**) Triglycerids contents in serum. Green color means NCD, red color for HFD, red hatching or arrows for O-1602 administration, blue hatching or arrows for CID16020046 administration. Results are presented as the means ± SDs of 5 mice per group. * *p* < 0.05, ** *p* < 0.01, *** *p* < 0.001 vs. the NCD group; # *p* < 0.05, ## *p* < 0.01, vs. O-1602 group.

To determine the therapeutic potential of GPR55, CID16020046 was administrated in 4-week high-fat diet-fed mice or in 4-week (3 times for a week) O-1602 treated mice for 5 days in the last week (Figure 7A).

Hepatic steatosis was induced by both high-fat diet feeding and O-1602 treatment (Figure 7B,C). CID16020046 administration for the last week reduced the lipid accumulation in livers in high-fat diet fed mice but not in O-1602-treated mice, which were judged by oil red O staining (Figure 7B,C). Furthermore, CID16020046 administration also reduced the ratio of liver/body weight significantly in high-fat diet fed mice (Figure 7D). On the other hand, the increased serum triglycerides levels by O-1602 administration was reduced by CID16020046 administration but was not blunted in high-fat diet-fed mice (Figure 7E).

## 3. Discussion

**Figure 8:**
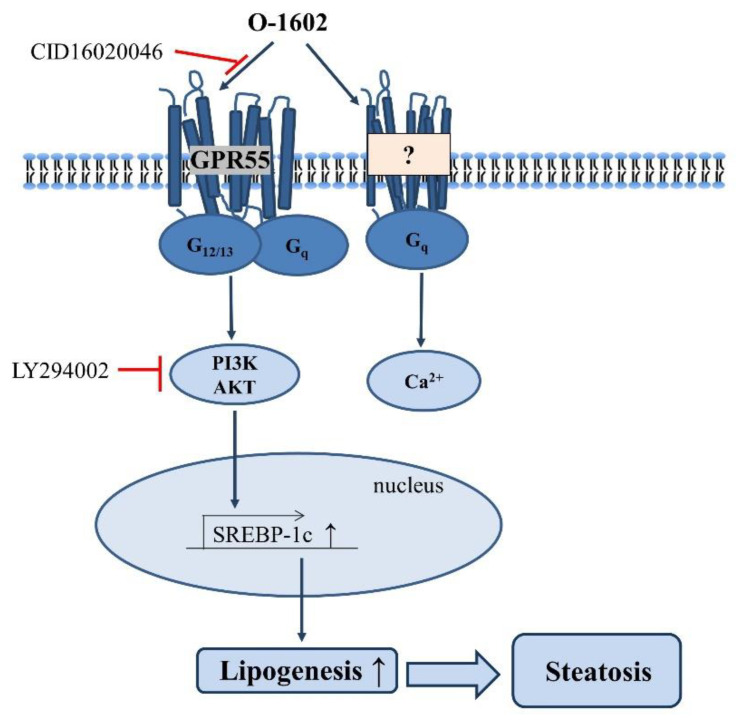
Proposed signaling pathway and mechanism of O-1602 on lipid accumulation in hepatocytes. O-1602 activation of GPR55 induces steatosis by inducing SREBP-1c through activation of PI3K/AKT and O-1602 induces Ca^2+^ increase in a GPR55-independent manner in hepatocytes.

The present study reports GPR55–dependent fat accumulation in hepatocytes and steatosis in the liver. Four key findings are reported. First, O-1602 induced lipid accumulation in Hep3B cells via GPR55. Second, O-1602-induced lipid accumulation was mediated by the expression of lipid-synthesizing SREBP-1c via the PI3K and Akt signaling pathways. Third, high-fat diet feeding increased the levels of lysophosphatidylinositols, the endogenous ligands of GPR55 in the liver. Fourth, the high-fat diet feeding in vivo induced lipid accumulation in the liver, which could be reversed by administration of CID16020046 (Figure 8). 

Recently, Fondevila et al. reported increased expression levels of liver GPR55 in human patients with non-alcoholic fatty liver diseases [11]. In several animal models of non-alcoholic hepatic steatosis and steatohepatitis, GPR55 was found to increase lipid content by inducing de novo fatty acid synthesis and decreasing fatty acid β oxidation, which is consistent with our results [11]. Serum levels of lysophosphatidylinositol species of 16:0, 18:1, and 18:1 isomer were found to be higher in patients with non-alcoholic steatohepatitis than in those with only steatosis [11]. The increased levels of lysophosphatidylinositols might contribute to the development of non-alcoholic hepatic steatohepatitis through the activation of GPR55 in hepatic stellate cells [11]. Our measurement of lysophosphatidylinositols also suggests that high-fat diets increase the levels of endogenous GPR55 agonists in the liver, which may activate GPR55 in hepatocytes and stellate cells, resulting in the development of hepatic steatosis and steatohepatitis. Indeed, in the present study, high-fat diet-fed mice showed the hepatic steatosis, which was reversed by CID16020046 administration, implying that increased lysophosphatidylinositols may be a cause of hepatic steatosis in over-nutrition conditions. O-1602 administration in vivo induced significant hepatic steatosis by itself but mild. The O-1602-induced hepatic steatosis was not reversed by administration of CID16020046. Considering the degree of hepatic steatosis induced by O-1602 was not severe as compared to high-fat diet feeding and CID16020046 administration duration and dose were fixed as one week and 1 mg/kg, further investigation is necessary.

Because insulin and insulin-like growth factors enhance not only glucose metabolism but also differentiation and survival in hepatocytes through the PI3K/Akt pathway [12,13], it is also important to consider the effects of GPR55 signaling through PI3K/Akt on insulin signaling on the metabolism, differentiation, and survival. Further investigation is necessary to clarify the cross-interaction betweeen insulin signaling and lysophosphatidylinositol signaling.

In this study, we found O-1602-induced Ca^2+^ increase in Hep3B cells in a GPR55-independent manner. GPR55-mediated Ca^2+^ increase has been observed in other cell types like HEK293 cells and mouse pancreatic β cells [14,15]. O-1602-induced Ca^2+^ increase was observed in isolated pancreatic β cells from wild-type mice but not from GPR55 gene-deficient mice [15], which proved O-1602-induced Ca^2+^ increase is mediated through GPR55 activation in pancreatic β cells. However, O-1602-induced Ca^2+^ increase in 3T3-L1 adipocytes was observed, but involvement of GPR55 was not assessed [16]. Therefore, it is necessary to verify carefully whether GPR55 is involved in Ca^2+^ increase in other cell types, because O-1602 may cause Ca^2+^ increase GPR55-independently as like Hep3B cells.

GPR55 in visceral and subcutaneous adipose tissue has been implicated in human obesity, and lysophosphatidylinositols increase the expression of lipogenic genes (fatty acid synthase and acetyl CoA carboxylase) and promote adipocyte differentiation by increasing PPARγ expression in visceral adipose tissues [4]. Conversely, these findings indicate that activation of GPR55 by increased lysophosphatidylinositols in hepatocytes and adipocytes results in the accumulation of fats and is associated with human obesity and metabolic disorders, suggesting the therapeutic potential of GPR55 in obesity-related diseases.

## 4. Materials and Methods

### 4.1. Materials

O-1602 and CID16020046 were purchased from Tocris Bioscience (Bristol, UK). Trimethylsilyldiazomethane (TMSD), methyl tert-butyl ether (MTBE), butylated hydroxytoluene (BHT), and ammonium acetate were obtained from Sigma-Aldrich (St. Louis, MO, USA). Lysophosphatidylinositol 17:1 (>99%) was obtained from Avanti Polar Lipids (Alabaster, AL, USA). All solvents for chromatography were liquid chromatography-mass spectrometry grade (Fisher Scientific, Pittsburgh, PA, USA). 

### 4.2. Cell Culture and Treatment

Human Hep3B hepatocytes were obtained from the American Type Culture Collection (ATCC, Manassas, VA, USA). Hep3B cells were maintained in Dulbecco’s modified Eagle medium with high glucose (Welgene, Daegu, Korea) with 10% (*v/v*) fetal bovine serum, 100 units/mL penicillin, 50 µg/mL streptomycin at 37 °C in a humidified atmosphere containing 5% CO_2_. Cells were seeded onto 6-well culture plates and allowed to adhere overnight (18 h).

### 4.3. Oil Red O Staining

Oil red O staining was performed according to a previously described method [17]. Briefly, cells were fixed with 10% formalin for 15 min at room temperature and then rinsed with PBS. The slides were immersed in Oil red O working solution for 1 h. After rinsing in tap water, slides were counterstained with hematoxylin for 5 s, rinsed with tap water, and mounted with an aqueous mounting medium.

### 4.4. Measurement of [Ca^2+^]_i_ Concentrations

Hep3B cells were trypsin-digested, allowed to sediment, resuspended in Hepes-buffered medium (HBM), consisting of 20 mM Hepes (pH 7.4), 103 mM NaCl, 4.8 mM KCl, 1.2 mM KH_2_PO_4_, 1.2 mM MgSO_4_, 0.5 mM CaCl_2_, 25 mM NaHCO_3_, 15 mM glucose, and 0.1% bovine serum albumin (fatty acid free), and then incubated for 40 min with 5 μM of Fura 2-AM. [Ca^2+^]_i_ levels were estimated by measuring changes in Fura 2 fluorescence using an emission wavelength of 510 nm and excitation wavelengths of 340 nm and 380 nm every 0.1 s using a F4500 fluorescence spectrophotometer (Hitachi, Japan). The ratios of fluorescence intensities (λ340/λ380) at these two wavelengths were used as a surrogate of [Ca^2+^]_i_, as previously described [18].

### 4.5. MTT Cytotoxicity Assay

Hep3B cells (4 × 10^5^ cells per well) were plated in 48-well flasks and starved for 24 h in DMEM containing 10% FBS. The cells were treated with O-1602 at the indicated concentrations for 24 h. Thirty microliters of 3-(4,5-dimethyl-2-thiazolyl)2,5-diphenyl-2H-tetrazolium bromide (MTT, 5 mg/mL) was added to the cell cultures and cultured for an additional 4 h in a humidified atmosphere. The cell culture media containing cells were collected and centrifuged, the supernatants were carefully removed, and the pellets were resuspended in 0.5 mL of DMSO:EtOH (1:1) solution and shaken for 10 min. Absorbance was measured at 570 nm by a SpectraCount microplate reader (Packard Instrument Co., Meriden, IL, USA); the optical density (OD) of untreated cells was defined as 100%.

### 4.6. Reverse Transcription-PCR

Total RNA was isolated from cells using Trizol reagent (Invitrogen, Waltham, MA, USA). RNA concentrations were determined by a NanoDrop ND-1000 spectrophotometer. One microgram of RNA was used for transcription, which was performed with the Promega ImProm-II Reverse Transicription System (Madision, WI, USA), according to the manufacturer’s protocol. Synthesized cDNA products and primers for each gene were used for PCR using Promega Go-Taq DNA polymerase (Madision, WI, USA). Specific primers for β-actin (sense 5′-CAC CAC ACC TTC TAC AAT GAG CTG-3′, antisense 5′-GAG GAG CAA TGA TCT TGA TCT TCA TT-3′), GPR55 (sense 5′-ATT ATG CTG CCA CCT CCA TC-3′ antisense 5′-TGA AGC AGA TGG TGA AGA CG-3′) were used to amplify gene fragments. PCR product aliquots (7 μL) were electrophoresed in 1.2% agarose gels and stained with nucleic acid gel stain (Real Biotech, Taiwan) [19].

### 4.7. Western Blot

Hep3B cells were harvested and resuspended in a lysis buffer. Protein content was determined using a BCA protein assay kit (Thermo scientific, Rockford, IL, USA) according to the manufacturer’s protocol. Cell lysates (30 µg protein) were separated by 8% SDS-PAGE, electrophoretically transferred to nitrocellulose paper, blocked with 5% skim milk, and then incubated with specific primary antibodies recognizing SREBP-1c (Santa Cruz Biotechnology, CA, USA) or Akt (pan), p-Akt (S473), β-actin (Cell Signaling Technology, Danvers, MA, USA) at 4 °C overnight. Blots were incubated with HRP-conjugated secondary antibody (Cell Signaling Technology, Danvers, MA, USA) and subsequently developed with ECL detection reagents [20]. Luminescence was detected using a ChemiDoc Touch Imaging System (BioRad, Hercules, CA, USA), followed by analysis with ImageLab software (BioRad).

### 4.8. Measurement of Triglycerides

Lipids were extracted with methanol/chloroform (1:2; *v/v*). The solvent was evaporated in 60°C, and the lipids were resuspended in deionized water. Triglyceride levels were determined using a commercial kit from Asan Pharm (Chungcheong, South Korea).

### 4.9. High Fat Diet Feeding

Male C57BL/6 mice were obtained from Daehan Biolink (DBL, Seoul, Korea). The mice had ad libitum access to water and food in the laboratory animal facility at PNU. Eight-week-old mice were randomly divided into 2 groups for lysophosphatidylinositol analysis (Figure 6). Control C57BL/6 mice (*n* = 5) were fed with a normal chow diet for 4 weeks while high-fat diet C57BL/6 mice (*n* = 5) were fed a synthetic diet supplemented with 60% *w/w*) fat (HFD, Efeed, Korea) for 4 weeks (Figure 6). The other sets were randomly divided into 5 groups for fatty liver induction and CID16020046 treatment (Figure 7): Control C57BL/6 mice fed a normal chow diet for 4 weeks (*n* = 5), high-fat diet C57BL/6 mice fed a synthetic diet supplemented with 60% (*w/w*) fat (HFD, Efeed, Korea) for 4 weeks (*n* = 5), high-fat diet C57BL/6 mice treated with CID16020046 treatment for the last week (*n* = 5), C57BL/6 mice treated with O-1602 (1 mg/kg, i.p. injection three times per week) for 4 weeks (*n* = 5), and C57BL/6 mice treated with O-1602 for 4 weeks plus CID16020046 (1 mg/kg, i.p. injection, five consecutive days for the last week) (*n* = 5) (Figure 7). The animal protocol used in this study was reviewed and approved by the PNU Institutional Animal Care Committee with respect to the ethics of the procedures and animal care (PNU-20192335).

### 4.10. Lysophosphatidylinositol Extraction and LC-MS/MS Analysis

Lysophosphatidylinositols were extracted from mouse liver according to the Matyash method with slight modifications [21]. In brief, lyophilized frozen liver was homogenized (30 frequency/min) 3 times for 5 min using a mixer mill (MM400, Retsch, Haan, Germany). Homogenized liver sample (5 mg) was added to the mixture solvent along with methanol and water (75:25, 400 μL, *v/v*) containing 0.1% BHT. Subsequently, 1.0 mL of MTBE was added and vortexed with vortex mixer for 1 hour in room temperature. Phase separation was induced by adding 250 μL of water. After mixing and centrifugation (14,000× *g*, 4 °C, 15 min), the upper (organic) phase was collected and dried under vacuum. For LPI analysis, dried supernatants were reconstituted in 100 μL methanol:chloroform (9:1, v/v) containing LPI 17:1 (internal standard, IS). After drying, it was resuspended in 500 μL MTBE and derivatized with 50 μL 2 M trimethylsilydiazomethane (TMSD) in hexane at 37 °C for 30 min. After incubation, glacial acetic acid (3 μL) and lower phase of MEBE:methanol:water mixture (500 μL, 100:30:25, *v/v/v*) were added and centrifuged (14,000× *g*, 5 min, 4 °C). The upper phase was collected, dried under vacuum, and reconstituted in 100 μL methanol:chloroform (9:1, *v/v*).

LPIs were analyzed by liquid chromatography-tandem mass spectrometry (LC-MS/MS, LC-MS 8060, Shimadzu, Kyoto, Japan). The LC-MS/MS system was equipped with a Kinetex C18 column (100 × 2.1 mm i.d., 2.6 μm particle size; Phenomenex, Torrance, CA, USA). The mobile phase consisted of 10 mM ammonium acetate in water/methanol (A, 5/5, *v/v*) and 10 mM ammonium acetate in methanol/isopropanol (B, 1/1, *v/v*). The gradient elution condition was as follows: 25% B (0 min), 80% B (4–7 min), and 25% B (10 min). Five microliters of derivatized sample was injected, and the flow rate was maintained at 0.2 mL/min. The mass operating conditions were as follows: desolvation temperature, 250 °C; heat block temperature, 400 °C; spray voltage. 4 kV; drying gas (N_2_) flow rate, 15 L/min; nebulizing gas (N_2_) flow rate, 3 L/min; collision gas (argon) pressure, 230 kPa; and detector voltage, 1.66 kV. Quantitation was conducted in selected reaction monitoring (SRM) modes with the precursor-to-product ion transition for each LPI (Table 1).

### 4.11. Statistical Analysis

All results were expressed as mean ± SD. Differences among groups were tested for statistical significance using analysis of variance (ANOVA) followed by Turkey’s post hoc test. A *p* value < 0.05 was considered statistically significant.

## Figures and Tables

**Table 1:** Selected reaction monitoring condition for the analysis of lysophosphatidylinositol (LPI) and internal standard (IS).

No.	Compound	Adduct	Precursor Ion (*m/z*)	Product Ion (*m/z*)
1	LPI 16:1	[M+H^+^]	585.3	311.3
2	LPI 16:0		587.3	313.3
3	LPI 18:3		609.3	335.3
4	LPI 18:2		611.3	337.3
5	LPI 18:1		613.3	339.3
6	LPI 18:0		615.3	341.3
7	LPI 20:5		633.3	359.3
8	LPI 20:4		635.3	361.3
9	LPI 20:3		637.3	363.3
10	LPI 20:2		639.3	365.3
11	LPI 20:1		641.3	367.3
12	LPI 20:0		643.3	369.3
13	LPI 22:6		659.3	385.3
14	LPI 17:1 (IS)		599.3	325.3
